# C9ORF72 expansion in a family with bipolar disorder

**DOI:** 10.1111/bdi.12063

**Published:** 2013-04-01

**Authors:** Miriam H Meisler, Adrienne E Grant, Julie M Jones, Guy M Lenk, Fang He, Peter K Todd, Masoud Kamali, Roger L Albin, Andrew P Lieberman, Scott A Langenecker, Melvin G McInnis

**Affiliations:** a Department of Human Genetics, University of Michigan School of MedicineAnn Arbor, MI, USA; b Department of Neurology, University of Michigan School of MedicineAnn Arbor, MI, USA; c Department of Psychiatry, University of Michigan School of MedicineAnn Arbor, MI, USA; d University of Michigan Depression CenterAnn Arbor, MI, USA; e Geriatrics Research, Education and Clinical Center, VAAAHSAnn Arbor, MI, USA; f Michigan Alzheimer Disease CenterAnn Arbor, MI, USA; g Department of Pathology, University of MichiganAnn Arbor, MI, USA

**Keywords:** atypical bipolar, C9ORF72, repeat expansion

## Abstract

**Objective:**

To investigate the role in bipolar disorder of the C9ORF72 hexanucleotide repeat expansion responsible for frontotemporal lobe dementia and amyotrophic lateral sclerosis.

**Methods:**

Eighty-nine subjects from a previously described panel of individuals with bipolar disorder ascertained for genetic studies were screened to detect expansion of the C9ORF72 repeat. One two-generation family with bipolar disorder and an expanded repeat was characterized in depth using molecular diagnostics, imaging, histopathology, and neurological and neuropsychological evaluation.

**Results:**

One proband, with the typical clinical presentation of bipolar disorder, carried an expanded C9ORF72 allele of heterogeneous length between 14 and 20 kilobases (kb) as assessed by Southern blot. The expanded allele was inherited from a parent with atypical, late onset clinical features of bipolar disorder, who subsequently progressed to frontotemporal lobe dementia. The expansion in peripheral blood of the parent ranged from 8.5 to 20 kb. Cultured lymphoblastoid cells from this parent exhibited a homogeneous expansion of only 8.5 kb.

**Conclusions:**

The disease course in the two generations described here demonstrates that expansion of the C9ORF72 may be associated with a form of bipolar disorder that presents clinically with classic phenomenology and progression to neurodegenerative disease. The frequency in our bipolar disorder cohort was only 1%, indicating that C9ORF72 is not a major contributor to bipolar disorder. DNA from cultured cells may be biased towards shorter repeats and nonrepresentative of the endogenous C9ORF72 expansion.

Expansion of the GGGGCC hexanucleotide within the C9ORF72 gene to several thousand tandem repeats results in frontotemporal lobe dementia (FTD) and amyotrophic lateral sclerosis (ALS), referred to as C9ALSFTD 1–3. C9ORF72 expansions are responsible for approximately 40% of familial ALS, 7% of sporadic ALS, and 25% of familial FTD in European populations 4, 5. The clinical heterogeneity caused by the C9ORF72 expansion extends to corticobasal and ataxia syndromes 6. More than 40% of patients with C9ALSFTD present with psychiatric disorders 7–9.

Population studies of several thousand individuals described repeat lengths below 30 in unaffected individuals. The frequency of expanded alleles in unaffected individuals was 3/856 (0.3%) in one study 4 and 5/2585 (0.2%) in another 5. Southern blot analysis of the expanded C9ORF72 repeat reveals a heterogeneous distribution of expansion length with a *smeared* appearance indicative of somatic instability 10. Intergenerational repeat instability and genetic anticipation are characteristic of other repeat expansion disorders 11 and may occur in C9ALSFTD 2. The role of C9ORF72 in bipolar disease has not previously been examined. Here, we describe the results of a small genetic screen. We have characterized a small family with bipolar disorder with apparent intergenerational expansion of the C9ORF72 repeat length and anticipation in the onset of psychopathology.

## Materials and methods

### Clinical samples

A panel of individuals with bipolar disorder, ascertained for genetics studies, was assessed clinically and neuropsychologically as previously described 12. Screening for the expansion in C9ORF72 was carried out based on the reports of a high frequency of psychiatric disturbances in individuals with expansions 7. The University of Michigan Institutional Review Board (Ann Arbor, MI, USA) approved the use of these samples for genetic studies of bipolar disorder.

### Polymerase chain reaction (PCR) assays

**Fig. 1:**
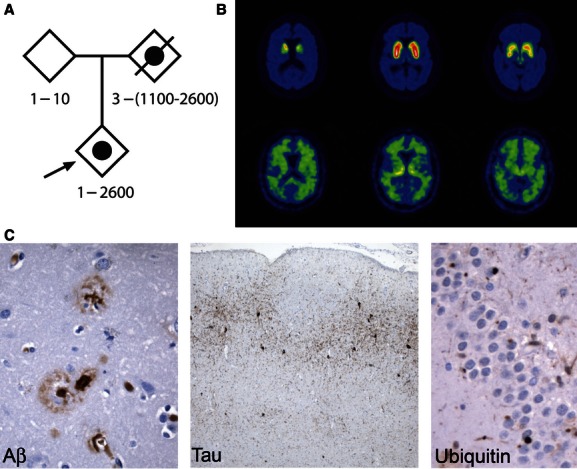
Clinical features of the family. (**A**) Pedigree demonstrating inheritance of bipolar disorder (solid circle) by the proband (arrow). Frontotemporal lobe dementia was diagnosed in the deceased parent. Genotypes represent hexanucleotide repeat numbers based on data in Figure 2. (**B**) Positron emission tomography imaging of the affected parent. Dopamine terminal tracer [^11^C] 3-alkyl-dihydrotetrabenazine (DTBZ) binding (top row) is modestly reduced, inconsistent with Lewy body dementia; [^11^C]PiB binding (bottom row) exhibits a subcortical white matter distribution without the prominent neocortical labeling seen in Alzheimer's disease and some cases of Lewy body dementia 14. (**C**) Histopathology of the affected parent. Left panel: Aβ stain highlights neuritic plaques and amyloid deposition in a small vessel in the parietal cortex (200×). Middle panel: Tau pathology in the entorhinal cortex (AT8 stain, 100×). Right panel: Ubiquitin-positive, tau-, transactive response DNA-binding protein 43-, and α-synuclein-negative cytoplasmic inclusions in dentate gyrus (400×).

Repeat-primed PCR was carried out as described 1 with two modifications: (i) 1.6 M betaine (Sigma Chemicals, St. Louis, MO, USA) 13 was freshly dissolved and added to each reaction immediately before PCR; and (ii) touch-down PCR was followed by an additional 27 cycles at 97°C for 35 sec, 54.5°C for 35 sec, and 68°C for 3 min + 20 sec per cycle, terminating at 68°C for 10 min. Short alleles (1–10 repeats) were amplified with forward primer F1 (5′ CCG CAG CCT GTA GCA AGC) located upstream of the repeat (Fig. 1A) and reverse primer FAM-R1 from the repeat-primed assay. Fragment sizes were determined on an ABI 3730 Sequencer. Additional details are provided in the *Supplementary materials*.

### Southern blot

The 241-base pair (bp) hybridization probe 3 located upstream of C9ORF72 was amplified from genomic DNA, gel purified, doubly radiolabeled with ^32^P-dCTP plus ^32^P-dATP (Perkin Elmer, Shelton, CT, USA) using the Prime-a Gene Labeling System (Promega, Madison, WI, USA), and separated from unincorporated nucleotides by centrifugation through a Sephadex-G50 column. A sample of 10 μg of genomic DNA was digested overnight with XbaI in the presence of 4 mM spermidine. Restriction fragments were separated by electrophoresis on 0.8% agarose gels with 1× tris-borate-EDTA buffer and transferred to Zetaprobe GT filters (BioRad Labs, Hercules, CA, USA). Hybridization with the radiolabeled probe was carried out in 0.5 M sodium phosphate, 7% sodium dodecyl sulfate (SDS), pH 7.2 at 65°C overnight. Filters were washed at 65°C for 15 min twice in solutions containing 0.1% SDS: 2× saline–sodium citrate (SSC), 1× SSC, 0.2× SSC, and 0.1× SSC. BioMax-MS film (Kodak, Rochester, NY, USA) was exposed with an intensifying screen at −80°C for two days. The use of double-labeled probes improved the sensitivity of the assay.

### Immunohistochemistry

Formalin-fixed, paraffin embedded sections were prepared from the middle frontal gyrus, anterior cingulate gyrus, insular cortex, superior and middle temporal lobes, inferior parietal cortex, visual cortex, thalamus and subthalamic nucleus, amygdala, nucleus basalis, hippocampus at the level of the lateral geniculate nucleus, midbrain at the level of the red nucleus, pons, medulla oblongata, cerebellar folia, and dentate nucleus. Sections were stained by hematoxylin and eosin, and selected sections were stained by Bielschowsky silver stain and by the immunoperoxidase method using a Ventana automated stainer. Primary antibodies were to glial fibrillary acidic protein (#20334; DAKO, Carpinteria, CA, USA), tau (AT8; Fisher, Pittsburgh, PA, USA), transactive response DNA-binding protein 43 (#10782-2-AP; Proteintech Group, Chicago, IL, USA), ubiquitin (UB1; Chemicon, Temecula, CA, USA), b-amyloid (6F-3D; Leica, Buffalo Grove, IL, USA) and a-synuclein (LB509; Invitrogen, Carlsbad, CA, USA).

## Results

### Screen for C9ORF72 expansion in a bipolar disorder cohort

Eighty-nine DNA samples were selected from a previously described panel of individuals with bipolar disorder ascertained for genetic studies 12. The selected samples were enriched for early onset and neurocognitive abnormalities. Screening was carried out by repeat-primed PCR. Three individuals who were homozygous for a short allele of C9ORF72, and six individuals with unclear PCR results, were further tested by PCR with flanking markers as described below. One patient with an expanded allele of C9ORF72 is characterized here.

### Clinical features

The family is represented in Figure 1A and is of Northern European ancestry. The 35-year-old bipolar proband has the typical clinical pattern of a bipolar disorder and was diagnosed following hospitalization due to an acute manic episode at 25 years of age. The disorder is successfully managed with lithium and an antidepressant. Current assessment was carried out using the Diagnostic Interview for Genetic Studies (DIGS) with DSM-IV criteria 12. Neuropsychiatric testing reported normal executive and memory ability, with evidence, discernible only by laboratory-based testing, of slightly disrupted fine motor functioning (*Supplementary Fig*. 1). Additional clinical description is provided in the *Supplementary information*.

The proband's unaffected parent has good mental health. The affected parent has a history of hospitalization for bipolar disorder beginning at age 62. This individual has an atypical clinical pattern that includes many attributes of mood irregularities that are common within the bipolar spectrum 14 (*detailed in the Supplementary information*). FTD was subsequently diagnosed after the development of apathy, progressive language dysfunction, progressive memory impairment, and gait disorder. Neurological evaluation at age 66 reported parkinsonian features. The Mini-Mental State Examination score was 19. Evaluation with the National Alzheimer Coordinating Center Uniform Data Set battery revealed impairments of attention, executive function, verbal fluency, and memory (Clinical Dementia Rating sum of boxes score = 11.5). Positron emission tomography imaging with the dopamine terminal ligand [^11^C] 3-alkyl-dihydrotetrabenazine (DTBZ) and the amyloid ligand [^11^C]PiB revealed a mild reduction in striatal dopamine terminals and no evidence of neocortical Aβ amyloid deposition (Fig. 1B) 14. Magnetic resonance imaging revealed modest anterior temporal atrophy. The affected parent's condition progressed to death at age 69. At autopsy, the brain weighed 1210 g and showed mild-to-moderate frontal and temporal lobe atrophy. Microscopic evaluation revealed findings of both argyrophilic grain disease and Alzheimer's disease. Alzheimer's disease neuropathological changes (A1, B1, C2) (Fig. 1C, left panel) were low level 15, and were accompanied by the presence of marked tau pathology in the anteromedial temporal lobe (Fig. 1C, middle panel). Although no TDP-43 inclusions were identified, neurons in the frontal and temporal cortex and dentate gyrus contained ubiquitinated cytoplasmic aggregates (Fig. 1C, right panel).

### C9ORF72 repeat detection

**Fig. 2:**
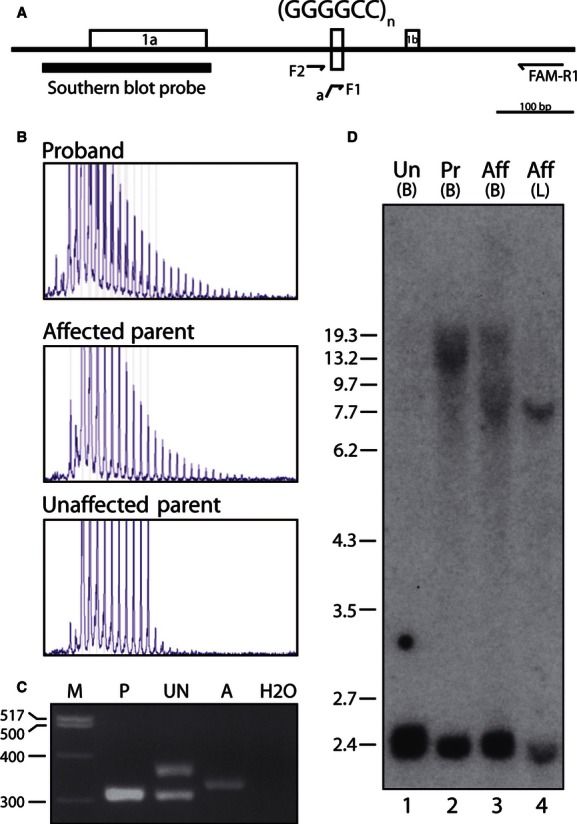
Molecular characterization of C9ORF72 in family members. (**A**) Noncoding exons 1a and 1b of C9ORF72 indicating locations of PCR primers and Southern blot probe. The 716 bp region shown corresponds to chromosome 9p21 from 27573248 bp (at right) to 27753964 bp (at left) (GRCh37/hg19). The position of the anchor probe sequence at the 5′ end of primer F1 is marked ‘a’. (**B**) Repeat-primed polymerase chain reaction (PCR) using primers F1, FAM-R1, and a, detected the saw-tooth pattern indicative of an expanded hexanucleotide repeat in the proband and affected parent. The sharp drop-off of peak height, characteristic of short alleles, in seen in the unaffected parent. (**C**) Amplification of the short hexanucleotide repeat alleles using PCR primers F2 and FAM-R1 reveals two different short alleles in the unaffected parent and a single short allele in the affected parent and proband. (**D**) Southern blot of genomic DNA from peripheral blood (lanes 1–3) demonstrates expanded alleles of 14–20 kb in the proband and 8.5–20 kb in the affected parent. Lymphoblastoid cells from the affected parent exhibit shorter repeat lengths. A gel containing radiolabeled molecular weight standards is presented in *Supplementary Figure*. 2. Aff = affected parent; B = peripheral blood; bp = base pair; L = cultured lymphoblastoid cells; Pr = proband; Un = unaffected parent.

The PCR primers and hybridization probe used for molecular analysis are shown in Figure 2A. Repeat-primed PCR of genomic DNA revealed the saw-tooth pattern of products indicative of an expanded repeat in genomic DNA from the proband and the affected parent (Fig. 1B). The unaffected parent's amplification products demonstrated a sharp drop-off in length consistent with nonexpanded alleles. This was confirmed by amplification of two short alleles of 306 and 363 bp from the unaffected parent, corresponding to one and ten hexanucleotide copies, using the flanking markers F2 and FAM-R1 (Fig. 2C). The proband inherited the one-copy allele from the unaffected parent. The affected parent contains a single short allele that was not inherited by the proband. (The presence of the single short allele is consistent either with homozygosity or with the presence of a second allele that is expanded and not detectable with these primers.)

### Length of C9ORF72 expanded repeats

Southern blot analysis of genomic DNA isolated from peripheral blood revealed expanded hexanucleotide repeat alleles in the proband and the affected parent (Fig. 2D, lanes 1–3). The 2.3 and 2.4 kb unexpanded alleles in the unaffected parent are also visible on short exposure (*Supplementary Fig*. 2). The proband carries a short allele of 2.3 kb and a heterogeneous expanded allele with average length of ∼18 kb, which corresponds to ∼2600 hexanucleotide copies. Size heterogeneity is typical for C9ORF72 expansions and is thought to reflect somatic instability of the expanded repeat. The affected parent also carries a heterogeneous expanded allele with a broader size distribution extending from 8.5 to 20 kb, with predominance of shorter expansions. Comparison of the size distribution of expansions between proband and affected parent demonstrates a greater representation of longer expansions in the proband.

### Expansion length in cultured lymphoblastoid cells

The most commonly studied source of genomic DNA for Southern blots of the C9ORF72 expansion is cultured lymphoblast cell lines. However, comparison of blood and cultured lymphoblasts from the affected parent revealed a large difference (Fig. 2D, lanes 3 and 4). In contrast with the broad size distribution in genomic DNA from whole blood, the lymphoblast cultures are greatly enriched for the short, 8.5 kb expansion length. The data suggest that there may be selection during culture for cells containing short repeats, and indicate that repeat lengths in lymphoblast DNA may be of limited diagnostic value.

## Discussion

We describe an expanded C9ORF72 repeat in a family with bipolar disorder and FTD. During intergenerational transmission from an affected parent, there was an apparent increase in the average length of the repeat. This affected parent had FTD that was initially diagnosed as late-onset bipolar disease. The parent exhibited a broad distribution of repeat length between 8.5 and 20 kb, whereas the proband with early onset carried a longer distribution between 14 and 20 kb. The initial presentation in both generations is bipolar disorder, suggesting an etiological relationship with the C9ORF72 repeat expansion. Published examples of individuals with bipolar disorder who later developed FTD have not included genetic characterization 16, 17. Intergenerational expansion of repeated elements in other genes is associated with genetic anticipation and increased disease severity in successive generations 11. We previously presented evidence of clinical anticipation in bipolar disorder 18. In the currently studied family, there appears to be a relationship between the age of onset and average repeat length, suggestive of genetic anticipation. The proband has typical bipolar phenomenology with fine motor abnormalities discernible only in the laboratory. This raises questions regarding the contribution of the C9ORF72 expansion to phenotypic heterogeneity within bipolar disorder and the relationship between fine motor dysfunction, mood disorder, the progression of neurodegenerative disorders, and genetic anticipation.

The most easily accessed and commonly used source of genomic DNA for Southern blots of C9ORF72 is lymphoblastoid cell lines. We found a significant difference between the expansion profile in DNA from peripheral blood and lymphoblasts cultured from the same sample. Shorter expansions may confer a growth advantage during culture, as has been demonstrated in myotonic dystrophy, where the somatic instability of a CTG repeat expansion in tissues is poorly represented in lymphoblastoid cell lines 19, 20. There is likely to be a complex relationship between the length of the C9ORF72 expansion observed in the laboratory and the clinical features of the patient, due to confounding factors that include age and tissue source, somatic instability, and cell growth conditions. It seems increasingly likely that the degree of expansion in accessible peripheral tissues may not reflect the genetic pathology present in the brain and nervous system.

Increased size distribution of the C9ORF72 repeat with transmission from an affected parent was recently described in an FTD family that included two affected siblings with different repeat profiles in peripheral blood 10. The reliability of extrapolating neuronal genotypes from peripheral blood or lymphocytes remains to be determined. In the future, comparisons between multiple tissues will be important for understanding the mechanism and extent of somatic variation in repeat length. Somatic variation may be an important factor in the clinical heterogeneity in FTD–ALS families, where longer expansions in motor neurons might lead to ALS, whereas expansion in cortical neurons might result in FTD. Similarly, the length of the expanded trinucleotide repeat in the muscle of patients with myotonic dystrophy can be considerably longer than in blood or lymphoblastoid cells 21.

The minimal C9ORF72 expansion required for pathogenicity is not known, and the penetrance of disease in individuals carrying expanded alleles remains to be determined 3. One study 5 reported a 50% likelihood of disease manifestation by age 57 and almost 100% by age 80. Few studies have examined the transmission of expanded C9ORF72alleles 8. Earlier onset in later generations of families with the C9ORF72 expansion has been described 2, 21. RNA-mediated pathology is suggested by a dependence on repeat length, but reduced RNA expression from the expanded allele has also been described 2. Further studies will be needed to determine the relative contributions of repeat length to RNA toxicity, protein toxicity, and reduced gene expression.

Our findings suggest a possible etiological relationship between the C9ORF72 expansion and disease progression from bipolar disorder to FTD. The affected parent has a history of lifetime temperamental instability reminiscent of the bipolar spectrum 14 and was hospitalized with incapacitating manic features in the seventh decade of life. The offspring with the larger average repeat expansion developed bipolar disorder in the third decade. In the future, it will be of great interest to examine C9ORF72 in two-generation families with bipolar disorder with or without dementia, in those with atypical cognitive and personality profiles, and in families with other neuropsychiatric syndromes demonstrating clinical anticipation. This approach may provide an insight into the variety of symptoms within the bipolar spectrum of disease, including intergenerational variability of clinical phenomena 14. The C9ORF72 expansion could also have an impact on early brain development, as suggested for the trinucleotide repeat in Huntington's disease 22. Screening by PCR can detect the repeat expansion, but Southern blots will be required to demonstrate genetic anticipation and determine the relationship between repeat length and clinical presentation. The frequency of the C9ORF72 expansion in our bipolar disorder cohort was approximately 1%, indicating that this is unlikely to be a common cause of bipolar disease. Analysis of larger cohorts of affected individuals will be needed to better define the contribution of the C9ORF72 repeat expansions to bipolar disorder.
