# Supplementary material for: C9ORF72 expansion in a family with bipolar disorder
Source: Bipolar Disord. 2013 Apr 1;15(3):326–32. doi: 10.1111/bdi.12063 (PMC3660726; doi:10.1111/bdi.12063)
Supplement: Supplementary file 1 [file bdi0015-0326-SD1.docx]

**C9ORF72 expansion in a family with bipolar disorder**

MH Meisler^1,2^, AE Grant^1^, JM Jones^1^, GM Lenk^1^, F He^2^, PK Todd^2^,

M Kamali^3,7^, RL Albin^2,5,6^, AP Lieberman^4,6^, SA Langenecker^3,7^, MG McInnis^3,7^

Departments of ^1^Human Genetics, ^2^Neurology, ^3^Psychiatry, ^4^Pathology, University of Michigan

School of Medicine, ^5^Geriatrics Research, Education and Clinical Center, VAAAHS,

^6^Michigan Alzheimer Disease Center, ^7^University of Michigan Depression Center, Ann Arbor MI

**Supplementary Methods**

**Repeat and flanking PCR assay for the C9ORF72 hexanucleotide**. Repeat-primed PCR of 50-100 ng of genomic DNA was carried out by a modification of the method of Renton et al (2011). We used 3.75 U Expand Long Template Enzyme Mix and 10% Expand Long Template Buffer 2 (Roche) with 5% DMSO (Fisher), 1.6 M betaine (Sigma) (Saluto et al, 2005), 0.35 mM dNTPs and 0.5-1 uM primer concentration in a 30 ul reaction volume. Betaine was freshly dissolved and added separately to each sample immediately before PCR. After incubation at 98°C for 10 min, 14 cycles of touchdown PCR were carried out with reduction of temperature from 68°C to 54°C by 1°C per cycle as described rather than the 2°C per cycle as in Renton et al (2011). This was followed by an additional 27 cycles of 97°C for 35 seconds, 54.5°C for 35 seconds, and 68°C for 3 min + 20 seconds per cycle, terminating with incubation at 68°C for 10 min. Short alleles (1-10 repeats) were amplified with forward primer F1 (5' CCG CAG CCT GTA GCA AGC) located upstream of the hexanucleotide and reverse primer FAM-R1 from the repeat-primed assay (see Figure 1A, text, for positions of primers). Repeat PCR products were separated on an ABI 3730 Sequencer (Applied Biosystems) in the University of Michigan Sequencing Core and analyzed with GeneMarker software (SoftGenetics LLC, State College, PA).

**Supplementary clinical description of the proband**

The proband appears to have a typical clinical pattern of a bipolar disorder. He is presently 37-years old and was diagnosed in his mid-twenties with bipolar I disorder following hospitalization due to an acute manic episode. Three weeks prior to this admission he experienced a progressive increase in ideas of reference, intense religious experiences with visions, hypergraphia, pressured speech, hyper-social activity with shopping (which normally he did not enjoy), decreased need for sleep and racing thoughts. Follow up management included successful lithium maintenance. Five years later he was seen in an academic ER for evaluation for hypomania having been recently discharged from a weeklong admission for a mixed affective state. A significantly diminished need for sleep, reved speech and irritable mood was noted and a referral to the local specialty mood clinic was made. Depressive episodes were present historically, and dated back to puberty when there were reports of suicidal rumination and at least one gesture. In recent years sub-threshold depressive periods were interspersed with hypomanic, irritable energy bursts of varying intensity and duration. In the decade since the initial diagnosis, there was a documented response to lithium with a prevailing need for an antidepressant due to a low baseline mood following mood stabilization with lithium. The medical history is unremarkable. The proband is a married responsible parent with marked achievements in employment. A strong therapeutic alliance with the clinical care provider along with a stable medication regime (lithium and bupropion) provides strong support for the challenges and occasional turbulence in family and personal life. The clinical diagnostic interview using the Diagnostic Interview for Genetic Studies (DIGS) had verified the diagnosis of bipolar disorder according to DSM IV TR criteria. Neuropsychiatric testing reported normal executive and memory ability, but significant evidence of disrupted fine motor functioning was found. The proband continues to do well clinically and personally and is adherent to treatment recommendations.

**Supplementary clinical description of the affected parent**

The affected parent exhibited an atypical clinical pattern for bipolar disorder and had been brought to the research clinic by the proband with concerns about bipolar disorder that had been diagnosed during 3 recent community hospitalizations. There had been limited response to various pharmacological interventions that included mood stabilizers, antipsychotic and antidepressant medications. Mood symptoms included exaggerated and energized grandiose behaviors that were provocative and unmanageable for the family. Over the parent’s lifetime inspirations and energy levels appear to have fluctuated in course and intensity. An intended vocation was derailed following a period of interpersonal provocative conflicts resulting in expulsion from a chosen field. The affected parent subsequently finished a career in manufacturing. Evaluation was completed using the DIGS and additional self-report and psychological assessments. The initial assessment identified features that were consistent with a probable history of mania, and was considered to be hypomanic and grandiose at the time of assessment, with loud and disinhibited descriptions of his achievements and plans.

The neuropsychiatric assessment was significantly impaired and with the history of late onset mania an organic basis for current disorder was considered. Additional assessment and imaging evaluation was consistent with a dementia diagnosis, most likely frontotemporal dementia (FTD).

***Supplementary Figures***

**Atypical Cognitive and Personality Profile:**

The differences between the cognitive and personality profiles for the proband and the affected parent, compared with typical profiles for individuals affected with bipolar disease are represented in *Supplementary Figure 1*. The proband and affected parent demonstrates less personality deviation and relatively greater fine motor disruption compared to the BP group. The proband does not demonstrate the cognitive and personality profile typified by the BP group (orange bar). The affected parent demonstrates excessive impairment, likely to be related to the late onset diagnosis of FTD and the C90RF72 expansion. These differences suggest that the C90RF72 expansion may contribute to phenotypic heterogeneity in BP disease.

***Supplementary Figure 1.* Cognitive and Personality Profiles**


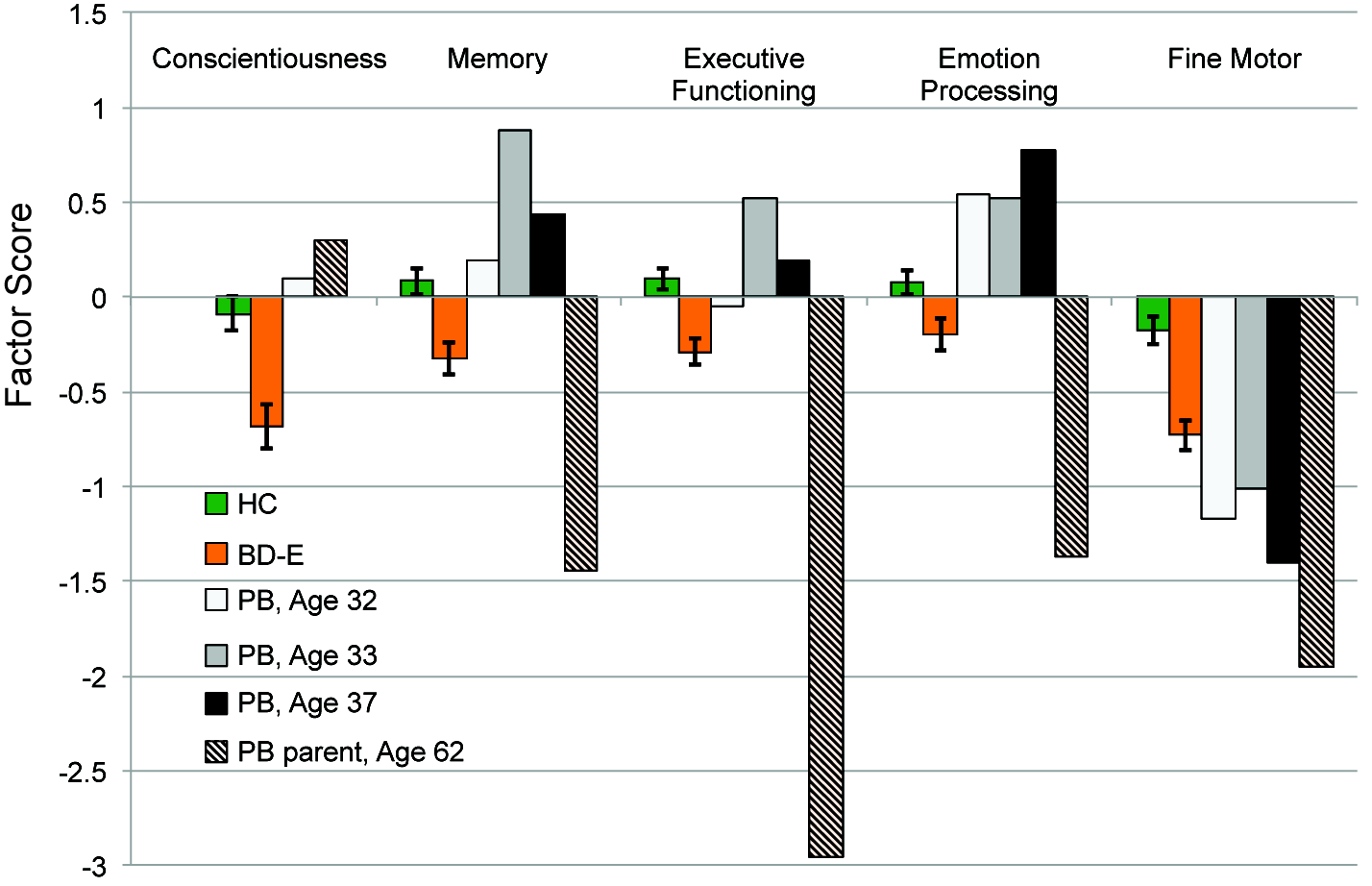


Green bars, 150 healthy control individuals; orange bars, 135 BP individuals in the euthymic (E) state, taken from the PLBS 500 sample (Langenecker et al., 2010, Ryan et al., 2012). Conscientiousness is a personality trait (Costa and McCrae 1992) that is significantly lower in E-BP individuals relative to healthy control comparison subjects. Memory included a collapsed representation of visual and auditory memory factors. Executive functioning is a collapsed representation of the four EF factors (Verbal Fluency and Processing Speed, Processing Speed with Interference Resolution, Conceptual Reasoning and Set-Shifting, and Inhibitory Control). Emotion processing is based upon auditory and visual emotion perception. Fine motor refers to manual dexterity in quickly placing circular metal pegs into a pegboard.

***Supplementary Figure 2.*** Southern blot from Figure 1 showing expanded region of blot and molecular weight markers. Markers and samples were run on the same gel, transferred, then cut to probe the membrane with sample lanes, and arranged together for exposure. Short exposure of the blot allowing resolution of the smaller alleles is shown below. Pr = proband; Aff = affected parent; Unaff = unaffected parent; B = blood; L = lymphoblast cells. M1, M2, molecular weight markers.

**
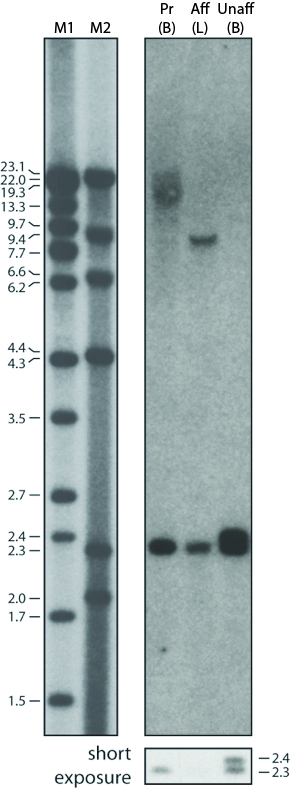
**

**References**

Renton AE, Majounie E, Waite A et al*.* A hexanucleotide repeat expansion in C9ORF72 is the cause of chromosome 9p21-linked ALS-FTD. Neuron 2011; 72: 257–268.

Saluto A, Brussino A, Tassone F et al. An enhanced polymerase chain reaction assay to detect pre- and full mutation alleles of the fragile X mental retardation 1 gene. J Mol Diagn 2005; 7: 605–612.

Costa PT, McCrae RR. Revised NEO-PI: Professional Manual. Odessa: Psychological Assessment Resources, 1992.

Langenecker SA, Saunders EF, Kade AM et al*.* Intermediate: cognitive phenotypes in bipolar disorder. J Affect Disord 2010; 122: 285–293.

Ryan KA, Vederman AC, McFadden EM et al. Differential executive functioning performance by phase of bipolar disorder. Bipolar Disord 2012; 14: 527–536.
